# Relationships Between Exposure to Gestational Diabetes Treatment and Neonatal Anthropometry: Evidence from the Born in Bradford (BiB) Cohort

**DOI:** 10.1007/s10995-023-03851-w

**Published:** 2023-11-29

**Authors:** Gilberte Martine-Edith, William Johnson, Emily S. Petherick

**Affiliations:** School of Sport, Exercise and Health Sciences, Loughborough University, Epinal Way, Loughborough, LE11 3TU UK

**Keywords:** Born in Bradford, Gestational diabetes, Neonatal anthropometry, Metformin

## Abstract

**Objectives:**

To examine the relationships between gestational diabetes mellitus (GDM) treatment and neonatal anthropometry.

**Methods:**

Covariate-adjusted multivariable linear regression analyses were used in 9907 offspring of the Born in Bradford cohort. GDM treatment type (lifestyle changes advice only, lifestyle changes and insulin or lifestyle changes and metformin) was the exposure, offspring born to mothers without GDM the control, and birth weight, head, mid-arm and abdominal circumference, and subscapular and triceps skinfold thickness the outcomes.

**Results:**

Lower birth weight in offspring exposed to insulin (− 117.2 g (95% CI − 173.8, − 60.7)) and metformin (− 200.3 g (− 328.5, − 72.1)) compared to offspring not exposed to GDM was partly attributed to lower gestational age at birth and greater proportion of Pakistani mothers in the treatment groups. Higher subscapular skinfolds in offspring exposed to treatment compared to those not exposed to GDM was partly attributed to higher maternal glucose concentrations at diagnosis. In fully adjusted analyses, offspring exposed to GDM treatment had lower weight, smaller abdominal circumference and skinfolds at birth than those not exposed to GDM. Metformin exposure was associated with smaller offspring mid-arm circumference (− 0.3 cm (− 0.6, − 0.07)) than insulin exposure in fully adjusted models with no other differences found.

**Conclusions for Practice:**

Offspring exposed to GDM treatment were lighter and smaller at birth than those not exposed to GDM. Metformin-exposed offspring had largely comparable birth anthropometric characteristics to those exposed to insulin.

**Supplementary Information:**

The online version contains supplementary material available at 10.1007/s10995-023-03851-w.

## Significance

*What is already known on this subject?* Offspring exposed to untreated GDM are more likely to have higher weight and adiposity at birth in comparison to offspring not exposed to GDM.

*What this study adds?* Offspring exposed to GDM treatment of any type (lifestyle changes advice, insulin or metformin) were lighter and less adipose than offspring not exposed to GDM. Metformin and insulin-exposed offspring had comparable anthropometric outcomes.

**Supplementary Information:**

The online version contains supplementary material available at 10.1007/s10995-023-03851-w.

## Introduction

Gestational diabetes mellitus (GDM) is a pregnancy complication characterised by an impaired glucose tolerance (Kaaja & Rönnemaa, 2008). In the United Kingdom (UK), GDM prevalence is approximately 4.4%, a total of 30,625 births each year (National Institute for Health and Care, 2015). GDM poses an important risk for maternal and neonatal health. Foetal exposure to GDM is associated with fat accumulation and growth acceleration in utero, thus, if GDM is left untreated, offspring of women with GDM (OGDM) are more likely to be heavier and more adipose at birth than offspring not exposed to GDM (Logan et al., 2017). In the long term, OGDM have an increased obesity and diabetes risk (Tieu et al., 2017).

Clinical management of GDM aims to maintain maternal glucose levels within healthy ranges, reducing the risk of adverse neonatal outcomes (Tarry-Adkins et al., 2020). Lifestyle changes are initially recommended and if these are insufficient to restore euglycaemia, pharmacotherapy is started (National Institute for Health and Care Excellence (NICE), 2015). Insulin has been historically the first-line pharmacological treatment for GDM. Metformin was introduced as a cheaper and more easily administered alternative to insulin however, it has been associated with safety concerns for the foetus as metformin, unlike insulin, crosses the placenta (Lindsay & Loeken, 2017).

Previous research has investigated the associations between exposure to maternal GDM treatment and offspring birth outcomes. When comparing OGDM to offspring not exposed to GDM, GDM treatment effects remain unclear as recent studies showed that OGDM had lower birth weight and/or were less adipose than offspring not exposed to GDM (Crowther et al., 2005; Landon et al., 2009; Prentice et al., 2019), whilst others described that OGDM were heavier or more adipose than offspring not exposed to GDM despite maternal treatment (Baptiste-Roberts et al., 2012; Prentice et al., 2019). None of those studies however have stratified OGDM by maternal treatment type, it is therefore difficult to determine whether, in comparison to offspring not exposed to GDM, differences in neonatal anthropometric outcomes are specific to the type of treatment prescribed. Further, in the comparison of GDM pharmacological treatment types, it was demonstrated that metformin was a safe alternative to insulin based on evidence of delivery outcomes and neonatal complications (Barrett et al., 2013; Ijäs et al., 2011; Niromanesh et al., 2012; Rowan et al., 2008; Tertti et al., 2013) but in terms of neonatal anthropometry, the evidence to date is largely based on birth weight (Niromanesh et al., 2012; Rowan et al., 2008; Tertti et al., 2008). More accurate evidence regarding neonatal anthropometry, especially in ethnically diverse populations, could however be obtained from circumference measurements and skinfold thicknesses (West et al., 2015).

This study aimed to, in a UK multi-ethnic birth cohort, examine the associations between exposure to maternal GDM treatment and neonatal anthropometry by (1) comparing offspring not exposed to GDM to OGDM exposed to treatment (lifestyle changes advice only or lifestyle changes advice with insulin or metformin) and (2) comparing metformin-exposed offspring to insulin-exposed offspring.

## Methods

### Study

Born in Bradford (BiB) is a longitudinal prospective birth cohort study conducted between March 2007 and December 2010 at the Bradford Royal Infirmary (Wright et al., 2013). Bradford is one of the UK’s largest cities characterised by high deprivation levels and ethnic diversity (Wright et al., 2013). Recruitment was mainly performed at the time of GDM screening. This was offered between 26 and 28 weeks of pregnancy to all women booked for delivery (80% of women attended the appointment) using the 2 h 75 g oral glucose tolerance test (OGTT) (Wright et al., 2013). Data were collected on 12,453 women, 13,776 pregnancies and 13,858 children. Ethical approval for the study was provided by Bradford Research Ethics Committee (Ref 07/H1302/112). All participants provided informed written consent for the study.

### Sample

**Fig. 1:**
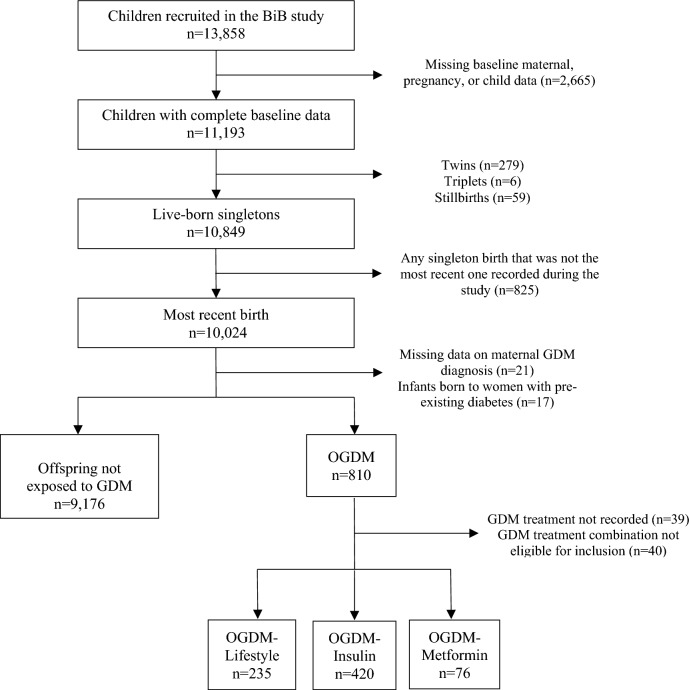
Flowchart of study participation

Our sample comprised 9,907 singleton infants with maternal, pregnancy and birth data (Fig. 1).

### Inclusion Criteria

If women had more than one singleton pregnancy during the study, only the most recently born infant was considered for inclusion. We included the following: (i) offspring not exposed to GDM, (ii) OGDM with maternal clinical records indicating that lifestyle change advice was received for GDM management (OGDM-Lifestyle), (iii) OGDM with records indicating maternal lifestyle change advice and insulin treatment of GDM (OGDM-Insulin) and (iv) OGDM with medical records specifying maternal lifestyle change advice and metformin treatment of GDM (OGDM-Metformin).

### Exclusion Criteria

Stillbirths, twins, and triplets were excluded. Infants born to women with GDM who received any other treatment combination were excluded as the numbers were insufficient to conduct meaningful analyses. Were also excluded infants of women with pre-existing diabetes or where type of GDM treatment was not recorded.

### Exposure to Maternal GDM Treatment

We considered four groups: offspring not exposed to GDM, OGDM-Lifestyle, OGDM-Insulin and OGDM-Metformin. GDM was diagnosed using the modified 1999 WHO criteria (fasting glucose concentration ≥ 6.1 mmol/L or 2 h post-load glucose ≥ 7.8 mmol/L) (World Health Organization, 1999). All women with GDM in our sample initially received lifestyle change advice by the clinical team. This consisted of recommendations for changes in dietary habits and a minimum of 30 min walking per day. If glucose targets (fasting plasma glucose: 4.0–5.5 mmol/L; 2 h postprandial: < 7.5 mmol/L) were not achieved by lifestyle changes, supplemental insulin injections alone were prescribed in the first part of the BiB study (04/2007–03/2009). After metformin introduction (04/2009), supplemental insulin injections and metformin tablets (850 mg/L, twice a day) were both pharmacological treatment options for GDM.

### Neonatal Outcomes

We examined six neonatal outcomes: birth weight, head, mid-arm and abdominal circumference, and subscapular and triceps skinfold thickness. Data were obtained from maternity hospital notes or electronic health records. Immediately after delivery, birth weight was measured with SECA digital scales. The remaining anthropometric measures were collected within 24 to 72 h following delivery by paediatricians, midwives, and research assistants. Using Lasso-o tapes (Harlow Printing Ltd, South Shields, UK), head circumference was calculated between the most anterior and posterior parts of the head and abdominal circumference was measured at the umbilical level. Subscapular and triceps skinfold thickness measurements were performed on the left side of the new-born with Tanner/Whitehouse Calipers (Holtain Ltd).

### Confounding Variables

Based on recent evidence of maternal characteristics associated with GDM pharmaceutical treatment in the BiB cohort (Martine-Edith et al., 2021) and previous BiB literature (West et al., 2013), we considered maternal body mass index (BMI), height, age at childbirth, ethnicity, parity, fasting and 2 h glucose concentrations at OGTT, smoking during pregnancy as confounding variables. Child sex, gestational age at delivery and route of birth were added to the model as competing exposures (Tennant et al., 2021). Age, parity (0, 1, 2 or 3 + children), smoking (yes/no) and ethnicity (White British (WB), Pakistani, Other) were self-reported by women at recruitment during interviewer-administered questionnaires. Ethnic groups were determined in accordance with the Office of National Statistics guidelines (Office for National Statistics, 2003). Maternal booking BMI was calculated from weight measured at pregnancy booking (10–14 weeks of gestation) using SECA digital scales and height at baseline (26–28 weeks of gestation) measured with Leicester Height Measure. A glucose oxidase method was used to estimate the fasting and 2 h post-load plasma glucose concentrations at OGTT. Gestational age at delivery and route of birth (vaginal or caesarean) were obtained from maternal health records.

### Statistical Analysis

Descriptive analysis was used to examine the characteristics of offspring not exposed to GDM, OGDM-Lifestyle, OGDM-Insulin and OGDM-Metformin. Categorical variables were presented as frequencies and percentages. Continuous variables were tested for normality and presented as mean and standard deviation (SD).

We used linear regression to explore the relationships between maternal GDM treatment and offspring birth weight, head, mid-arm and abdominal circumference, and subscapular and triceps skinfold thickness. We assessed these relationships firstly in a whole sample analysis by comparing OGDM-Lifestyle, OGDM-Insulin and OGDM-Metformin to offspring not exposed to GDM. Secondly, in a subsample analysis of OGDM, OGDM-Metformin were compared to OGDM-Insulin. For both analyses we built unadjusted and adjusted models for each outcome.

Lastly, we performed sensitivity analyses. We reproduced the whole sample analysis: (i) in participants with complete data on each outcome, exposure and confounding variable, (ii) stratified by ethnicity, (iii) stratified by route of birth and (iv), in the period after metformin introduction.

Analyses were conducted using Stata/SE software (Stata/SE 15 for Windows; StataCorp, College Station, TX, USA).

## Results

Of the 13,858 infants included in the BiB study, 9907 singleton live births met our study inclusion criteria. Our sample comprised 9176 offspring not exposed to GDM, 235 OGDM-Lifestyle, 420 OGDM-Insulin and 76 OGDM-Metformin (Fig. 1).

### Descriptive Analysis

Maternal age at childbirth was higher in OGDM than offspring not exposed to GDM (Table 1). Mean maternal BMI was the highest for OGDM-Insulin (29.2 (SD 6.3)) and OGDM-Metformin (29.6 (SD 6.2)). There were more Pakistani women in all OGDM groups than there was in offspring not exposed to GDM. Maternal post-load glucose concentrations at OGTT were higher for OGDM-Insulin (9.2 (SD 1.7)) and OGDM-Metformin (8.9 (SD 1.2)) than for offspring not exposed to GDM (5.4 (SD 1.0)). OGDM were born earlier and lighter than offspring not exposed to GDM. The prevalence of caesarean deliveries was higher among OGDM-Insulin (32.9%), OGDM-Metformin (31.6%), and OGDM-Lifestyle (25.5%) than offspring not exposed to GDM (21.2%).

**Table 1:** Maternal and offspring characteristics

	Offspring not exposed to GDM (n = 9176)	OGDM-Lifestyle (n = 235)	OGDM-Insulin (n = 420)	OGDM-Metformin (n = 76)	Missing n (%)
Maternal characteristics					
Age at childbirth (years), mean (SD)	27.9 (5.5)	30.0 (5.4)	32.0 (5.2)	31.1 (6.0)	0
Height (cm), mean (SD)	161.8 (6.4)	159.0 (6.4)	159.7 (6.5)	159.9 (5.8)	222 (2.2)
BMI atbooking(kg/m^2^), mean (SD)	25.9 (5.6)	25.8 (5.1)	29.2 (6.3)	29.6 (6.2)	492 (5.0)
Ethnic group, n (%)					18 (0.2)
White British	3,802 (41.5)	44 (18.9)	112 (26.7)	15 (19.7)	
Pakistani	3,952 (43.1)	143 (61.4)	238 (56.7)	54 (71.0)	
Other	1,406 (15.3)	46 (19.7)	70 (16.7)	7 (9.2)	
Parity, n (%)					362 (3.7)
0	3,581 (40.5)	85 (37.3)	120 (29.8)	21 (28.0)	
1	2,629 (29.7)	49 (21.5)	96 (23.8)	20 (26.7)	
2	1,475 (16.7)	41 (18.0)	74 (18.4)	21 (28.0)	
3+	1,154 (13.1)	53 (23.2)	113 (28.0)	13 (17.3)	
Smoking, n (%)					20 (0.2)
Yes	1,603 (17.5)	12 (5.1)	44 (10.5)	6 (7.9)	
No	7,554 (82.5)	223 (94.9)	375 (89.5)	70 (92.1)	
History of GDM, n (%)					0
Yes	31 (0.3)	12 (5.1)	47 (11.2)	7 (9.2)	
No	9,145 (99.7)	223 (94.9)	373 (88.8)	69 (90.8)	
Family history of diabetes, n (%)					0
Yes	2,124 (23.2)	140 (59.6)	279 (66.4)	46 (60.5)	
No	7,052 (76.8)	95 (40.4)	141 (33.6)	30 (39.5)	
Gestational age at OGTT (weeks), mean (SD)	26.3 (1.9)	26.8 (2.5)	26.0 (2.3)	26.2 (1.8)	396 (4.0)
Fasting glucose concentrations at OGTT (mmol/L), mean (SD)	4.4 (0.4)	4.9 (0.7)	5.4 (1.0)	5.0 (0.7)	399 (4.0)
2h post-load glucose concentrations at OGTT (mmol/L), mean (SD)	5.4 (1.0)	8.4 (1.2)	9.2 (1.7)	8.9 (1.2)	406 (4.1)
Gestational age at delivery (weeks), mean (SD)	39.6 (1.7)	39.0 (1.6)	38.1 (1.0)	38.3 (0.8)	0
Route of birth, n (%)					0
Vaginal	7,227 (78.8)	175 (74.5)	282 (67.1)	52 (68.4)	
Caesarean	1,949 (21.2)	60 (25.5)	138 (32.9)	24 (31.6)	
Offspring characteristics					
Child sex, n (%)					0
Female	4,462 (48.6)	110 (46.8)	206 (49.0)	33 (43.4)	
Male	4,714 (51.4)	125 (53.2)	214 (50.9)	43 (56.6)	
Birth weight (g), mean (SD)	3,255.0 (549.5)	3103.6 (514.9)	3,136.6 (491.7)	3,030.4 (415.5)	1 (<1)

### Whole Sample Analysis: Offspring not Exposed to GDM vs. OGDM Exposed to Treatment

Unadjusted analyses showed that, in comparison to offspring not exposed to GDM, offspring exposure to GDM treatment of any type was associated with lower weight, head, mid-arm and abdominal circumference and higher subscapular skinfold thickness at birth (Table 2).

**Table 2:** Associations between maternal GDM treatment and neonatal anthropometric outcomes

	n	Unadjusted coefficients (95%CI)	p	Adjusted coefficients* (95%CI)	p	Adjusted standardised coefficients*^†^
Birth weight (g)						
Offspring not exposed to GDM	8047	Reference		Reference		Reference
OGDM-Lifestyle	215	− 153.7 (− 227.0, − 80.4)	< 0.001	− 141.3 (− 200.4, − 82.2)	< 0.001	− 0.04
OGDM-Insulin	368	− 117.2 (− 173.8, − 60.7)	< 0.001	− 77.8 (− 130.5, − 25.2)	0.004	− 0.03
OGDM-Metformin	69	− 200.3 (− 328.5, − 72.1)	0.002	− 131.5 (− 230.1, − 33.0)	0.009	− 0.02
Head circumference (cm)						
Offspring not exposed to GDM	7394	Reference		Reference		Reference
OGDM-Lifestyle	202	− 0.1 (− 0.4, 0.07)	0.190	0.02 (− 0.2, 0.2)	0.868	0.002
OGDM-Insulin	341	− 0.3 (− 0.4, − 0.1)	0.001	0.1 (− 0.02, 0.3)	0.091	0.02
OGDM-Metformin	63	− 0.2 (− 0.6, 0.1)	0.233	0.2 (− 0.1, 0.5)	0.302	0.01
Mid-arm circumference (cm)						
Offspring not exposed to GDM	6979	Reference		Reference		Reference
OGDM-Lifestyle	194	− 0.3 (− 0.5, − 0.2)	< 0.001	− 0.3 (− 0.5, − 0.2)	< 0.001	− 0.05
OGDM-Insulin	335	− 0.2 (− 0.3, − 0.05)	0.005	− 0.1 (− 0.3, 0.01)	0.070	− 0.02
OGDM-Metformin	59	− 0.6 (− 0.9, − 0.3)	< 0.001	− 0.5 (− 0.8, -0.2)	0.001	− 0.04
Abdominal circumference (cm						
Offspring not exposed to GDM	6991	Reference		Reference		Reference
OGDM-Lifestyle	194	− 0.7 (− 1.0, − 0.3)	< 0.001	− 0.6 (− 1.0, − 0.2)	0.002	− 0.03
OGDM-Insulin	334	− 0.6 (− 0.8, − 0.3)	< 0.001	− 0.3 (− 0.6, 0.02)	0.066	− 0.02
OGDM-Metformin	59	− 1.0 (− 1.6, − 0.3)	0.005	− 0.6 (− 1.3, 0.003)	0.051	− 0.02
Subscapular skinfold thickness (mm)						
Offspring not exposed to GDM	5560	Reference		Reference		Reference
OGDM-Lifestyle	161	0.04 (− 0.1, 0.2)	0.642	− 0.2 (− 0.3, 0.006)	0.058	− 0.02
OGDM-Insulin	279	0.1 (0.02, 0.3)	0.019	− 0.2 (− 0.4, − 0.06)	0.006	− 0.04
OGDM-Metformin	51	0.01 (− 0.3, 0.3)	0.946	− 0.3 (− 0.6, 0.04)	0.086	− 0.02
Triceps skinfold thickness (mm)						
Offspring not exposed to GDM	5574	Reference		Reference		Reference
OGDM-Lifestyle	162	− 0.005 (− 0.2, 0.2)	0.951	− 0.2 (− 0.4, − 0.01)	0.036	− 0.03
OGDM-Insulin	280	0.2 (0.04, 0.3)	0.009	− 0.1 (− 0.3, 0.01)	0.072	− 0.03
OGDM-Metformin	51	− 0.1 (− 0.4, 0.2)	0.369	− 0.3 (− 0.6, − 0.05)	0.022	− 0.03

*All models are adjusted for maternal BMI, height, age, ethnicity, parity, fasting and 2h glucose concentrations at OGTT, smoking, child sex, gestational age at birth and route of birth

^†^Standardised coefficients are in units of SD

Birth weight estimates for OGDM-Insulin and OGDM-Metformin in comparison to offspring not exposed to GDM were − 117.2 g (95% CI -173.8, -60.7) and − 200.3 g (− 328.5, − 72.1) respectively, and these were changed to 161.2 g (113.9, 208.4) and 47.6 g (-58.1, 153.3) upon adjustment for gestational age at birth (Online Resource 1). Negative estimates for birth weight and abdominal circumference for all treatment types in comparison to offspring not exposed to GDM were attenuated following adjustments for maternal height and ethnicity (Online Resources 1 and 2). Positive estimates for subscapular skinfold thickness for OGDM-Lifestyle (0.04 mm (-0.1, 0.2)), OGDM-Insulin (0.1 mm (0.02, 0.3)) and OGDM-Metformin (0.01 mm (-0.3, 0.3)) compared to offspring not exposed to GDM became negative after adjusting for maternal glucose concentrations at OGTT (Online Resource 3).

Upon adjustment for all confounding variables, OGDM exposed to treatment of any kind were predicted to have lower weight, mid-arm and abdominal circumference, lower skinfold thicknesses and larger head circumference at birth than offspring not exposed to GDM (Table 2). The standardised coefficients were of similar magnitude for each GDM treatment across the outcomes.

### Subsample Analysis: OGDM-Insulin vs. OGDM-Metformin

Unadjusted analyses showed that OGDM-Metformin had smaller mid-arm circumference (-0.4 cm (− 0.7, − 0.1)) and lower triceps skinfold thickness (− 0.3 cm (− 0.6, 0.01)) than OGDM-Insulin (Table 3). The associations between metformin exposure and smaller mid-arm circumference remained negative (− 0.3 cm (− 0.6, − 0.07)) following adjustments for confounding. OGDM-Metformin and OGDM-Insulin did not differ in any other outcomes.

**Table 3:** Associations between metformin treatment and neonatal anthropometric outcomes

	n	Unadjusted coefficients (95%CI)	p	Adjusted coefficients* (95%CI)	p
Birth weight (g)					
OGDM-Insulin	368	Reference		Reference	
OGDM-Metformin	69	− 83.0 (− 208.2, 42.1)	0.193	− 84.2 (− 189.6, 21.1)	0.117
Head circumference (cm)					
OGDM-Insulin	341	Reference		Reference	
OGDM-Metformin	63	0.05 (− 0.3, 0.4)	0.791	− 0.08 (− 0.4, 0.2)	0.645
Mid-arm circumference (cm)					
OGDM-Insulin	335	Reference		Reference	
OGDM-Metformin	59	− 0.4 (− 0.7, − 0.1)	0.007	− 0.3 (− 0.6, − 0.07)	0.015
Abdominal circumference (cm)					
OGDM-Insulin	334	Reference		Reference	
OGDM-Metformin	59	− 0.4 (− 1.1, 0.3)	0.254	− 0.4 (− 1.1, 0.3)	0.232
Subscapular skinfold thickness (mm)					
OGDM-Insulin	279	Reference		Reference	
OGDM-Metformin	51	− 0.1 (− 0.5, 0.2)	0.392	− 0.07 (− 0.4, 0.2)	0.681
Triceps skinfold thickness (mm)					
OGDM-Insulin	280	Reference		Reference	
OGDM-Metformin	51	− 0.3 (− 0.6, 0.01)	0.061	− 0.2 (− 0.6, 0.07)	0.132

*All models are adjusted for maternal BMI, height, age, ethnicity, parity, fasting and 2h glucose concentrations at OGTT, smoking, child sex, gestational age at birth and route of birth

### Sensitivity Analyses

The magnitude and direction of the relationships between exposure to GDM treatment in fully adjusted analyses were mostly unchanged by the stratification by maternal ethnicity (Online Resource 4) and route of birth (Online Resource 5). Conducting the same analysis in participants with complete data on all outcomes, exposure and confounding variables did not change the direction of the associations (Online Resource 6). The analysis of OGDM born to women treated after metformin introduction highlighted similar associations with neonatal anthropometric outcomes to those of the main analysis (Online Resource 7).

## Discussion

This study showed, in unadjusted analyses, that OGDM exposed to treatment had lower weight and abdominal circumference than offspring not exposed to GDM, which could be partly attributed to a lower gestational age at birth and a greater proportion of Pakistani women in OGDM exposed to treatment. Higher subscapular skinfold thickness at birth in OGDM exposed to treatment compared to offspring not exposed to GDM in unadjusted analyses could be partly attributed to higher glucose concentrations at OGTT in the OGDM groups. Following adjustments for all confounding variables, OGDM exposed to treatment of any kind were predicted to have a lower weight, mid-arm and abdominal circumference, smaller skinfold thicknesses and larger head circumference at birth than offspring not exposed to GDM. The results further demonstrated that OGDM-Metformin were not significantly different from OGDM-Insulin except for a smaller mid-arm circumference in fully adjusted models.

Gestational age at delivery, maternal ethnicity and severity of hyperglycaemia individually contributed to the differences observed between OGDM exposed to treatment and offspring not exposed to GDM. This is consistent with previous research which has shown that after accounting for factors including gestational age, OGDM exposed to treatment (in the study’s most recent cohort) no longer had lower birth weight than offspring not exposed to GDM (Prentice et al., 2019). It is possible that the lower gestational age at birth observed in OGDM exposed to treatment compared to offspring not exposed to GDM may reflect clinical decisions favouring an earlier birth through caesarean section in GDM pregnancies as GDM is associated with risk of macrosomia and shoulder dystocia (Adams et al., 1998; Naylor et al., 1996). No previous studies have been able to clearly show the confounding effect of South Asian ethnicity on the associations between GDM treatment exposure and offspring anthropometry and this may be because of ethnicities that were not White had small sample sizes (Logan et al., 2016) or were broadly categorised into a single group (Prentice et al., 2019). It is hypothesised that, in our study, the large representation of Pakistani women, who have been shown to have offspring with lower birth weight than WB women (West et al., 2010, 2013), in the OGDM groups contributed to the smaller birth weight and abdominal circumference in OGDM compared to offspring not exposed to GDM. Lastly, previous BiB research has shown in unadjusted analyses that the odds of higher offspring adiposity (measured as sum of skinfolds > 90th percentile) at birth increased with maternal glucose levels (Farrar et al., 2015). Our results corroborate this as higher maternal glucose levels at OGTT in the OGDM groups than offspring not exposed to GDM contributed to the observed higher subscapular skinfold thickness in OGDM at birth.

Despite the individual contributions of gestational age at delivery, maternal ethnicity and severity of hyperglycaemia to our results, important differences in anthropometric characteristics remained between OGDM exposed to treatment and offspring not exposed to GDM after adjustments for all covariables simultaneously. Thus, it is likely that GDM treatment itself was associated with lower weight and adiposity in OGDM compared to offspring not exposed to GDM in the cohort. This was demonstrated in offspring of both WB and Pakistani women, and those born from vaginal and caesarean deliveries. Our results are in line with a study conducted in the UK in which a regression discontinuity analysis showed a reduction in birth weight (200 g) and lower odds of large-for-gestational age infants in women diagnosed with GDM and treated for GDM in comparison to women directly below the thresholds for GDM diagnosis (Tennant et al., 2022). It was suggested that the finding of smaller and/or lighter OGDM exposed to treatment than offspring not exposed to GDM in recent studies may be the result of the increasing use of metformin over the years (Prentice et al., 2019), as one of metformin’s mechanisms of action is believed to affect placental glucose transport (Tarry-Adkins et al., 2020). However, this remained uncertain as no studies to date had stratified their analyses by treatment type when comparing OGDM exposed to treatment to offspring not exposed to GDM. Our study therefore provides reassuring evidence for clinical practice that OGDM did not have higher weight or adiposity than offspring not exposed to GDM, regardless of whether women were treated with lifestyle changes advice alone, supplementary insulin or supplementary metformin.

Lastly, this study demonstrated that OGDM-Metformin were comparable to OGDM-Insulin for most anthropometric outcomes. This is in line with previous evidence suggesting that when compared to insulin, metformin treatment is not associated with any significant differences in neonatal anthropometry (Barrett et al., 2013; Ijäs et al., 2011; Rowan et al., 2008; Tertti et al., 2008, 2013). It was however found in this study that OGDM-Metformin had smaller arm circumference than OGDM-Insulin. This may suggest that metformin exposure is associated with less peripheral fat storage which could mean that more fat is stored abdominally (Tarry-Adkins et al., 2020), although no differences were found in abdominal circumference in the current study. Nironamesh et al. (2012) also found in a randomised controlled trial that OGDM-Metformin had smaller arms, in addition to lower birth weight and height, and smaller head and chest circumference at birth. Similarly, other studies have found that OGDM-Metformin had lower birth weight than OGDM-Insulin (Ainuddin et al., 2015; Hamadani et al., 2017). The differences between OGDM-Metformin and OGDM-Insulin were however limited to arm size in our study. Further research is therefore required to establish whether these differences change in childhood. This is especially important as there is evidence that OGDM-Metformin have larger arm circumference and subscapular and biceps skinfold thickness than OGDM-Insulin by 2 years of age, suggesting a healthier pattern of fat storage in early childhood in OGDM-Metformin, as less fat may be stored in the abdomen (Rowan et al., 2011).

Our study had three main strengths. Firstly, these results were based on a large number of OGDM stratified by maternal treatment type which allowed for the assessment of the associations between individual treatment types and neonatal anthropometry, compared to offspring not exposed to GDM. Secondly, evidence has been produced using a variety of anthropometric birth data, allowing for a greater understanding, beyond the differences in birth weight, of the associations between exposure to GDM treatment and offspring adiposity distribution at birth. Lastly, given the differences in the risk of GDM development and neonatal anthropometric characteristics between Pakistani and WB women, the current findings stratified by maternal ethnicity contribute to knowledge by showing in fully adjusted analyses that lower offspring weight and adiposity at birth for OGDM exposed to treatment were observed in both Pakistani and WB groups. Despite these strengths, the study was limited by the relatively low number of women treated with metformin and their offspring, as metformin was introduced in the last two years of the BiB study. Further, there was no data on maternal compliance to treatment which would have had an impact on neonatal outcomes.

To conclude, offspring exposure to GDM treatment of any type in the BiB cohort was associated with lower weight, mid-arm and abdominal circumference and smaller skinfolds at birth compared to offspring not exposed to GDM. There was limited evidence that metformin, compared to insulin, was associated with differences in anthropometric characteristics at birth.

### Supplementary Information

Below is the link to the electronic supplementary material.

Supplementary material 1 (DOCX 110.2 kb)
