# Supplementary material for: Relationships Between Exposure to Gestational Diabetes Treatment and Neonatal Anthropometry: Evidence from the Born in Bradford (BiB) Cohort
Source: Matern Child Health J. 2023 Nov 29;28(3):557–66. doi: 10.1007/s10995-023-03851-w (PMC10914642; doi:10.1007/s10995-023-03851-w)
Supplement: Supplementary file 1 — Supplementary material 1 (DOCX 110.2 kb) [file 10995_2023_3851_MOESM1_ESM.docx]

**Relationships between exposure to gestational diabetes treatment and neonatal anthropometry: Evidence from the Born in Bradford (BiB) cohort**

Gilberte Martine-Edith^a^, William Johnson^a^, Emily S Petherick^a*^

*^a^School of Sport, Exercise and Health Sciences, Loughborough University, Loughborough, UK*

Correspondence to: ^*^Emily Petherick, School of Sport, Exercise and Health Sciences, Loughborough University, Epinal Way, LE11 3TU, Loughborough, UK. E-mail: [e.petherick@lboro.ac.uk](mailto:e.petherick@lboro.ac.uk)

**Online Resource 1** Associations between GDM treatment and birth weight (g) with individual adjustments for confounding variables

| Confounding variable | n | Unadjusted coefficients  (95%CI) | Adjusted  coefficients  (95%CI) |
| --- | --- | --- | --- |
| **Maternal BMI**  No-GDM  OGDM-Lifestyle  OGDM-Insulin  OGDM-Metformin | 8,047  215  368  69 | Reference  -153.7 (-227.0, -80.4)  -117.2 (-173.8, -60.7)  -200.3 (-328.5, -72.1) | Reference  -155.9 (-227.7, -84.1)  -183.0 (-238.8, -127.2)  -271.3 (-397.1, -145.4) |
| **Maternal height**  No-GDM  OGDM-Lifestyle  OGDM-Insulin  OGDM-Metformin | 8,047  215  368  69 | Reference  -153.7 (-227.0, -80.4)  -117.2 (-173.8, -60.7)  -200.3 (-328.5, -72.1) | Reference  -101.0 (-172.4, -29.7)  -75.4 (-130.4, -20.3)  -165.0 (-289.7, -40.4) |
| **Maternal age at childbirth**  No-GDM  OGDM-Lifestyle  OGDM-Insulin  OGDM-Metformin | 8,047  215  368  69 | Reference  -153.7 (-227.0, -80.4)  -117.2 (-173.8, -60.7)  -200.3 (-328.5, -72.1) | Reference  -166.3 (-239.5, -93.1)  -145.7 (-202.7, -88.7)  -221.3 (-349.4, -93.3) |
| **Maternal ethnicity**  No-GDM  OGDM-Lifestyle  OGDM-Insulin  OGDM-Metformin | 8,047  215  368  69 | Reference  -153.7 (-227.0, -80.4)  -117.2 (-173.8, -60.7)  -200.3 (-328.5, -72.1) | Reference  -108.1 (-180.4, -35.9)  -86.0 (-141.7, -30.3)  -150.0 (-276.2, -23.7) |
| **Maternal parity**  No-GDM  OGDM-Lifestyle  OGDM-Insulin  OGDM-Metformin | 8,047  215  368  69 | Reference  -153.7 (-227.0, -80.4)  -117.2 (-173.8, -60.7)  -200.3 (-328.5, -72.1) | Reference  -154.3 (-227.4, -81.3)  -126.6 (-183.1, -70.0)  -212.0 (-339.7, -84.2) |
| **Fasting glucose at OGTT**  No-GDM  OGDM-Lifestyle  OGDM-Insulin  OGDM-Metformin | 8,047  215  368  69 | Reference  -153.7 (-227.0, -80.4)  -117.2 (-173.8, -60.7)  -200.3 (-328.5, -72.1) | Reference  -213.1 (-286.7, -139.5)  -247.2 (-308.2, -186.2)  -271.4 (-399.5, -143.4) |
| **2h post-load glucose at OGTT**  No-GDM  OGDM-Lifestyle  OGDM-Insulin  OGDM-Metformin | 8,047  215  368  69 | Reference  -153.7 (-227.0, -80.4)  -117.2 (-173.8, -60.7)  -200.3 (-328.5, -72.1) | Reference  -279.5 (-359.1, -199.8)  -276.8 (-346.1, -207.5)  -343.7 (-476.5, -210.9) |
| **Smoking during pregnancy**  No-GDM  OGDM-Lifestyle  OGDM-Insulin  OGDM-Metformin | 8,047  215  368  69 | Reference  -153.7 (-227.0, -80.4)  -117.2 (-173.8, -60.7)  -200.3 (-328.5, -72.1) | Reference  -165.7 (-239.0, -92.5)  -123.1 (-179.5, -66.6)  -210.0 (-338.0, -82.0) |
| **Child sex**  No-GDM  OGDM-Lifestyle  OGDM-Insulin  OGDM-Metformin | 8,047  215  368  69 | Reference  -153.7 (-227.0, -80.4)  -117.2 (-173.8, -60.7)  -200.3 (-328.5, -72.1) | Reference  -153.9 (-226.7, -81.0)  -117.2 (-173.3, -61.0)  -206.3 (-333.8, -78.9) |
| **Route of birth**  No-GDM  OGDM-Lifestyle  OGDM-Insulin  OGDM-Metformin | 8,047  215  368  69 | Reference  -153.7 (-227.0, -80.4)  -117.2 (-173.8, -60.7)  -200.3 (-328.5, -72.1) | Reference  -153.1 (-226.4, -79.8)  -115.9 (-172.6, -59.3)  -199.2 (-327.4, -71.0) |
| **Gestational age at delivery**  No-GDM  OGDM-Lifestyle  OGDM-Insulin  OGDM-Metformin | 8,047  215  368  69 | Reference  -153.7 (-227.0, -80.4)  -117.2 (-173.8, -60.7)  -200.3 (-328.5, -72.1) | Reference  -44.7 (-105.1, 15.6)  161.2 (113.9, 208.4)  47.6 (-58.1, 153.3) |
| **All confounding variables**  No-GDM  OGDM-Lifestyle  OGDM-Insulin  OGDM-Metformin | 8,047  215  368  69 | Reference  -153.7 (-227.0, -80.4)  -117.2 (-173.8, -60.7)  -200.3 (-328.5, -72.1) | Reference  -141.3 (-200.4, -82.2)  -77.8 (-130.5, -25.2)  -131.5 (-230.1, -33.0) |

BMI: body mass index; OGTT: oral glucose tolerance test

**Online Resource 2** Associations between GDM treatment and abdominal circumference (cm) at birth with individual adjustments for confounding variables

| Confounding variable | n | Unadjusted coefficients  (95%CI) | Adjusted  coefficients  (95%CI) |
| --- | --- | --- | --- |
| **Maternal BMI**  No-GDM  OGDM-Lifestyle  OGDM-Insulin  OGDM-Metformin | 6,991  194  334  59 | Reference  -0.7 (-1.0, -0.3)  -0.6 (-0.8, -0.3)  -1.0 (-1.6, -0.3) | Reference  -0.7 (-1.1, -0.3)  -0.8 (-1.1, -0.5)  -1.2 (-1.9, -0.5) |
| **Maternal height**  No-GDM  OGDM-Lifestyle  OGDM-Insulin  OGDM-Metformin | 6,991  194  334  59 | Reference  -0.7 (-1.0, -0.3)  -0.6 (-0.8, -0.3)  -1.0 (-1.6, -0.3) | Reference  -0.5 (-0.9, -0.1)  -0.4 (-0.7, -0.1)  -0.9 (-1.5, -0.2) |
| **Maternal age at childbirth**  No-GDM  OGDM-Lifestyle  OGDM-Insulin  OGDM-Metformin | 6,991  194  334  59 | Reference  -0.7 (-1.0, -0.3)  -0.6 (-0.8, -0.3)  -1.0 (-1.6, -0.3) | Reference  -0.7 (-1.1, -0.3)  -0.6 (-0.9, -0.3)  -1.0 (-1.7, -0.4) |
| **Maternal ethnicity**  No-GDM  OGDM-Lifestyle  OGDM-Insulin  OGDM-Metformin | 6,991  194  334  59 | Reference  -0.7 (-1.0, -0.3)  -0.6 (-0.8, -0.3)  -1.0 (-1.6, -0.3) | Reference  -0.4 (-0.8, -0.06)  -0.4 (-0.6, -0.09)  -0.7 (-1.4, -0.04) |
| **Maternal parity**  No-GDM  OGDM-Lifestyle  OGDM-Insulin  OGDM-Metformin | 6,991  194  334  59 | Reference  -0.7 (-1.0, -0.3)  -0.6 (-0.8, -0.3)  -1.0 (-1.6, -0.3) | Reference  -0.7 (-1.1, -0.3)  -0.6 (-0.9, -0.3)  -1.0 (-1.7, -0.3) |
| **Fasting glucose at OGTT**  No-GDM  OGDM-Lifestyle  OGDM-Insulin  OGDM-Metformin | 6,991  194  334  59 | Reference  -0.7 (-1.0, -0.3)  -0.6 (-0.8, -0.3)  -1.0 (-1.6, -0.3) | Reference  -0.8 (-1.1, -0.4)  -0.7 (-1.1, -0.4)  -1.1 (-1.8, -0.4) |
| **2h post-load glucose at OGTT**  No-GDM  OGDM-Lifestyle  OGDM-Insulin  OGDM-Metformin | 6,991  194  334  59 | Reference  -0.7 (-1.0, -0.3)  -0.6 (-0.8, -0.3)  -1.0 (-1.6, -0.3) | Reference  -1.0 (-1.4, -0.6)  -1.0 (-1.3, -0.6)  -1.4 (-2.1, -0.6) |
| **Smoking during pregnancy**  No-GDM  OGDM-Lifestyle  OGDM-Insulin  OGDM-Metformin | 6,991  194  334  59 | Reference  -0.7 (-1.0, -0.3)  -0.6 (-0.8, -0.3)  -1.0 (-1.6, -0.3) | Reference  -0.7 (-1.1, -0.3)  -0.5 (-0.8, -0.3)  -1.0 (-1.6, -0.3) |
| **Child sex**  No-GDM  OGDM-Lifestyle  OGDM-Insulin  OGDM-Metformin | 6,991  194  334  59 | Reference  -0.7 (-1.0, -0.3)  -0.6 (-0.8, -0.3)  -1.0 (-1.6, -0.3) | Reference  -0.7 (-1.1, -0.3)  -0.6 (-0.8, -0.3)  -1.0 (-1.7, -0.3) |
| **Route of birth**  No-GDM  OGDM-Lifestyle  OGDM-Insulin  OGDM-Metformin | 6,991  194  334  59 | Reference  -0.7 (-1.0, -0.3)  -0.6 (-0.8, -0.3)  -1.0 (-1.6, -0.3) | Reference  -0.7 (-1.1, -0.3)  -0.5 (-0.8, -0.3)  -1.0 (-1.6, -0.3) |
| **Gestational age at delivery**  No-GDM  OGDM-Lifestyle  OGDM-Insulin  OGDM-Metformin | 6,991  194  334  59 | Reference  -0.7 (-1.0, -0.3)  -0.6 (-0.8, -0.3)  -1.0 (-1.6, -0.3) | Reference  -0.4 (-0.7, -0.01)  0.3 (0.07, 0.6)  -0.1 (-0.7, 0.5) |
| **All confounding variables**  No-GDM  OGDM-Lifestyle  OGDM-Insulin  OGDM-Metformin | 6,991  194  334  59 | Reference  -0.7 (-1.0, -0.3)  -0.6 (-0.8, -0.3)  -1.0 (-1.6, -0.3) | Reference  -0.6 (-1.0, -0.2)  -0.3 (-0.6, 0.02)  -0.6 (-1.3, 0.003) |

BMI: body mass index; OGTT: oral glucose tolerance test

**Online Resource 3** Associations between GDM treatment and subscapular skinfold thickness (mm) at birth with individual adjustments for confounding variables

| Confounding variable | n | Unadjusted coefficients  (95%CI) | Adjusted  coefficients  (95%CI) |
| --- | --- | --- | --- |
| **Maternal BMI**  No-GDM  OGDM-Lifestyle  OGDM-Insulin  OGDM-Metformin | 5,560  161  279  51 | Reference  0.04 (-0.1, 0.2)  0.1 (0.02, 0.3)  0.01 (-0.3, 0.3) | Reference  0.04 (-0.1, 0.2)  0.04 (-0.08, 0.2)  -0.1 (-0.4, 0.2) |
| **Maternal height**  No-GDM  OGDM-Lifestyle  OGDM-Insulin  OGDM-Metformin | 5,560  161  279  51 | Reference  0.04 (-0.1, 0.2)  0.1 (0.02, 0.3)  0.01 (-0.3, 0.3) | Reference  0.08 (-0.09, 0.2)  0.2 (0.05, 0.3)  0.03 (-0.3, 0.3) |
| **Maternal age at childbirth**  No-GDM  OGDM-Lifestyle  OGDM-Insulin  OGDM-Metformin | 5,560  161  279  51 | Reference  0.04 (-0.1, 0.2)  0.1 (0.02, 0.3)  0.01 (-0.3, 0.3) | Reference  0.009 (-0.2, 0.2)  0.09 (-0.04, 0.2)  -0.04 (-0.3, 0.3) |
| **Maternal ethnicity**  No-GDM  OGDM-Lifestyle  OGDM-Insulin  OGDM-Metformin | 5,560  161  279  51 | Reference  0.04 (-0.1, 0.2)  0.1 (0.02, 0.3)  0.01 (-0.3, 0.3) | Reference  0.08 (-0.09, 0.2)  0.2 (0.05, 0.3)  0.06 (-0.2, 0.3) |
| **Maternal parity**  No-GDM  OGDM-Lifestyle  OGDM-Insulin  OGDM-Metformin | 5,560  161  279  51 | Reference  0.04 (-0.1, 0.2)  0.1 (0.02, 0.3)  0.01 (-0.3, 0.3) | Reference  0.04 (-0.1, 0.2)  0.1 (-0.0009, 0.3)  -0.03 (-0.3, 0.3) |
| **Fasting glucose at OGTT**  No-GDM  OGDM-Lifestyle  OGDM-Insulin  OGDM-Metformin | 5,560  161  279  51 | Reference  0.04 (-0.1, 0.2)  0.1 (0.02, 0.3)  0.01 (-0.3, 0.3) | Reference  -0.1 (-0.3, 0.07)  -0.1 (-0.3, -0.008)  -0.2 (-0.5, 0.1) |
| **2h post-load glucose at OGTT**  No-GDM  OGDM-Lifestyle  OGDM-Insulin  OGDM-Metformin | 5,560  161  279  51 | Reference  0.04 (-0.1, 0.2)  0.1 (0.02, 0.3)  0.01 (-0.3, 0.3) | Reference  -0.3 (-0.5, -0.1)  -0.3 (-0.4, -0.1)  -0.4 (-0.7, -0.09) |
| **Smoking during pregnancy**  No-GDM  OGDM-Lifestyle  OGDM-Insulin  OGDM-Metformin | 5,560  161  279  51 | Reference  0.04 (-0.1, 0.2)  0.1 (0.02, 0.3)  0.01 (-0.3, 0.3) | Reference  0.02 (-0.1, 0.2)  0.1 (0.02, 0.3)  -0.0008 (-0.3, 0.3) |
| **Child sex**  No-GDM  OGDM-Lifestyle  OGDM-Insulin  OGDM-Metformin | 5,560  161  279  51 | Reference  0.04 (-0.1, 0.2)  0.1 (0.02, 0.3)  0.01 (-0.3, 0.3) | Reference  0.04 (-0.1, 0.2)  0.2 (0.03, 0.3)  0.03 (-0.3, 0.3) |
| **Route of birth**  No-GDM  OGDM-Lifestyle  OGDM-Insulin  OGDM-Metformin | 5,560  161  279  51 | Reference  0.04 (-0.1, 0.2)  0.1 (0.02, 0.3)  0.01 (-0.3, 0.3) | Reference  0.03 (-0.1, 0.2)  0.1 (-0.0005, 0.3)  -0.01 (-0.3, 0.3) |
| **Gestational age at delivery**  No-GDM  OGDM-Lifestyle  OGDM-Insulin  OGDM-Metformin | 5,560  161  279  51 | Reference  0.04 (-0.1, 0.2)  0.1 (0.02, 0.3)  0.01 (-0.3, 0.3) | Reference  0.1 (-0.07, 0.3)  0.3 (0.2, 0.4)  0.2 (-0.1, 0.5) |
| **All confounding variables**  No-GDM  OGDM-Lifestyle  OGDM-Insulin  OGDM-Metformin | 5,560  161  279  51 | Reference  0.04 (-0.1, 0.2)  0.1 (0.02, 0.3)  0.01 (-0.3, 0.3) | Reference  -0.2 (-0.3, 0.006)  -0.2 (-0.4, -0.06)  -0.3 (-0.6, 0.04) |

BMI: body mass index; OGTT: oral glucose tolerance test

**Online Resource 4** Associations between maternal GDM treatment and neonatal outcomes stratified by maternal ethnicity

|  | **White British** | | | **Pakistani** | | |
| --- | --- | --- | --- | --- | --- | --- |
|  | **n** | **Adjusted coefficients***  **(95%CI)** | ***p*** | **n** | **Adjusted coefficients***  **(95%CI)** | ***p*** |
| **Birth weight (g)**  No-GDM  OGDM-Lifestyle  OGDM-Insulin  OGDM-Metformin | 3,360  40  98  14 | Reference  -161.3 (-292.6, -29.9)  -64.6 (-157.6, 28.5)  -125.5 (-341.9, 91.0) | 0.016  0.174  0.256 | 3,457  131  209  49 | Reference  -127.7 (-204.9, -50.6)  -92.6 (-165.5, -19.6)  -123.3 (-241.2, -5.5) | 0.001  0.013  0.040 |
| **Head circumference (cm)**  No-GDM  OGDM-Lifestyle  OGDM-Insulin  OGDM-Metformin | 3,089  38  92  13 | Reference  -0.09 (-0.5; 0.3)  0.3 (-0.03; 0.6)  0.3 (-0.4; 1.0) | 0.680  0.076  0.375 | 3,179  122  194  44 | Reference  0.1 (-0.1, 0.4)  0.1 (-0.09, 0.4)  0.1 (-0.2, 0.5) | 0.284  0.219  0.447 |
| **Mid-arm circumference (cm)**  No-GDM  OGDM-Lifestyle  OGDM-Insulin  OGDM-Metformin | 2,921  37  90  13 | Reference  -0.3 (-0.7, 0.0006)  -0.01 (-0.2, 0.2)  -0.3 (-0.8, 0.3) | 0.050  0.928  0.315 | 3,001  117  191  40 | Reference  -0.4 (-0.6, -0.2)  -0.3 (-0.5, -0.05)  -0.6 (-1.0, -0.3) | <0.001  0.014  <0.001 |
| **Abdominal circumference (cm)**  No-GDM  OGDM-Lifestyle  OGDM-Insulin  OGDM-Metformin | 2,921  37  89  13 | Reference  -0.6 (-1.5, 0.2)  -0.3 (-0.9, 0.2)  0.09 (-1.2, 1.4) | 0.122  0.239  0.890 | 3,013  116  191  40 | Reference  -0.7 (-1.2, -0.2)  -0.2 (-0.7, 0.2)  -0.8 (-1.5, 0.02) | 0.006  0.280  0.057 |
| **Subscapular skinfold thickness (cm)**  No-GDM  OGDM-Lifestyle  OGDM-Insulin  OGDM-Metformin | 2,196  28  73  10 | Reference  -0.2 (-0.6, 0.2)  -0.2 (-0.5, 0.1)  0.01 (-0.7, 0.7) | 0.292  0.223  0.972 | 2,516  101  163  36 | Reference  -0.3 (-0.5, -0.03)  -0.4 (-0.6, -0.1)  -0.4 (-0.8, -0.09) | 0.028  0.001  0.014 |
| **Triceps skinfold thickness (cm)**  No-GDM  OGDM-Lifestyle  OGDM-Insulin  OGDM-Metformin | 2,205  28  73  10 | Reference  -0.4 (-0.8, 0.04)  -0.2 (-0.5, 0.08)  -0.3 (-1.0, 0.3) | 0.079  0.164  0.340 | 2,521  101  164  36 | Reference  -0.2 (-0.4, 0.04)  -0.1 (-0.3, 0.07)  -0.4 (-0.8, -0.06) | 0.117  0.196  0.023 |

*All models are adjusted for maternal BMI, height, age, parity, fasting and 2h glucose concentrations at OGTT, smoking, child sex, gestational age at birth and route of birth

**Online Resource 5** Associations between maternal GDM treatment and neonatal outcomes stratified by route of birth

|  | **Vaginal birth** | | | **Caesarean birth** | | |
| --- | --- | --- | --- | --- | --- | --- |
|  | **n** | **Adjusted coefficients***  **(95%CI)** | ***p*** | **n** | **Adjusted coefficients***  **(95%CI)** | ***p*** |
| **Birth weight (g)**  No-GDM  OGDM-Lifestyle  OGDM-Insulin  OGDM-Metformin | 6,361  160  250  48 | Reference  -109.3 (-174.0, -44.6)  -109.2 (-168.1, -50.2)  -137.9 (-249.9, -25.9) | 0.001  <0.001  0.016 | 1,690  55  118  21 | Reference  -206.1 (-343.3, -69.0)  -16.9 (-132.4, 98.6)  -108.5 (-315.2, 98.1) | 0.003  0.774  0.303 |
| **Head circumference (cm)**  No-GDM  OGDM-Lifestyle  OGDM-Insulin  OGDM-Metformin | 5,869  153  232  43 | Reference  -0.05 (-0.2, 0.2)  0.06 (-0.1, 0.2)  0.1 (-0.3, 0.5) | 0.651  0.548  0.561 | 1,529  49  109  20 | Reference  0.2 (-0.2, 0.7)  0.4 (0.005, 0.7)  0.3 (-0.3, 1.0) | 0.284  0.047  0.356 |
| **Mid-arm circumference (cm)**  No-GDM  OGDM-Lifestyle  OGDM-Insulin  OGDM-Metformin | 5,549  147  228  40 | Reference  -0.4 (-0.6, -0.2)  -0.2 (-0.4, -0.08)  -0.6 (-0.9, -0.2) | <0.001  0.004  0.001 | 1,434  47  107  19 | Reference  -0.2 (-0.5, 0.2)  0.1 (-0.1, 0.4)  -0.2 (-0.7, 0.3) | 0.316  0.336  0.420 |
| **Abdominal circumference (cm)**  No-GDM  OGDM-Lifestyle  OGDM-Insulin  OGDM-Metformin | 5,558  148  227  40 | Reference  -0.6 (-1.0, -0.2)  -0.6 (-1.0, -0.2)  -0.4 (-1.2, 0.3) | 0.004  0.003  0.257 | 1,437  46  107  19 | Reference  -0.4 (-1.2, 0.4)  0.3 (-0.3, 1.0)  -0.9 (-2.1, 0.2) | 0.349  0.325  0.116 |
| **Subscapular skinfold thickness (cm)**  No-GDM  OGDM-Lifestyle  OGDM-Insulin  OGDM-Metformin | 4,355  121  190  35 | Reference  -0.1 (-0.3, 0.1)  -0.2 (-0.4, -0.02)  -0.3 (-0.6, 0.09) | 0.342  0.031  0.146 | 1,208  40  89  16 | Reference  -0.3 (-0.7, 0.06)  -0.3 (-0.6, 0.05)  -0.2 (-0.8, 0.3) | 0.091  0.091  0.428 |
| **Triceps skinfold thickness (cm)**  No-GDM  OGDM-Lifestyle  OGDM-Insulin  OGDM-Metformin | 4,364  122  191  35 | Reference  -0.08 (-0.3, 0.1)  -0.1 (-0.3, 0.03)  -0.4 (-0.8, -0.09) | 0.400  0.093  0.015 | 1,213  40  89  16 | Reference  -0.4 (-0.8, -0.05)  -0.1 (-0.4, 0.2)  -0.1 (-0.7, 0.5) | 0.026  0.491  0.703 |

*All models are adjusted for maternal BMI, height, age, ethnicity, parity, fasting and 2h glucose concentrations at OGTT, smoking, child sex and gestational age at birth

|  | **n** | **Adjusted coefficients* (95%CI)** | ***p*** |
| --- | --- | --- | --- |
| **Birth weight (g)**  No-GDM  OGDM-Lifestyle  OGDM-Insulin  OGDM-Metformin | 5,278  153  266  49 | Reference  -155.9 (-225.8, -85.9)  -144.4 (-207.0, -81.8)  -217.5 (-334.4, -100.5) | <0.001  <0.001  <0.001 |
| **Head circumference (cm)**  No-GDM  OGDM-Lifestyle  OGDM-Insulin  OGDM-Metformin | 5,278  153  266  49 | Reference  -0.007 (-0.2, 0.2)  0.03 (-0.2, 0.2)  0.1 (-0.3, 0.4) | 0.951  0.783  0.595 |
| **Mid-arm circumference (cm)**  No-GDM  OGDM-Lifestyle  OGDM-Insulin  OGDM-Metformin | 5,278  153  266  49 | Reference  -0.4 (-0.5, -0.2)  -0.2 (-0.4, -0.06)  -0.6 (-0.9, -0.3) | <0.001  0.007  <0.001 |
| **Abdominal circumference (cm)**  No-GDM  OGDM-Lifestyle  OGDM-Insulin  OGDM-Metformin | 5,278  153  266  49 | Reference  -0.6 (-1.1, -0.2)  -0.4 (-0.8, -0.06)  -1.0 (-1.7, -0.3) | 0.002  0.024  0.007 |
| **Subscapular skinfold thickness (cm)**  No-GDM  OGDM-Lifestyle  OGDM-Insulin  OGDM-Metformin | 5,278  153  266  49 | Reference  -0.2 (-0.4, 0.006)  -0.2 (-0.4, -0.05)  -0.2 (-0.5, 0.09) | 0.058  0.011  0.168 |
| **Triceps skinfold thickness (cm)**  No-GDM  OGDM-Lifestyle  OGDM-Insulin  OGDM-Metformin | 5,278  153  266  49 | Reference  -0.2 (-0.4, -0.01)  -0.1 (-0.3, 0.01)  -0.3 (-0.6, -0.02) | 0.036  0.071  0.036 |

**Online Resource 6** Associations between maternal GDM treatment and neonatal outcomes in women with data on all outcomes, exposure, and confounding variables

*All models are adjusted for maternal BMI, height, age, ethnicity, parity, fasting and 2h glucose concentrations at OGTT, smoking, child sex, gestational age at birth and route of birth

**Online Resource 7** Associations between maternal GDM treatment and neonatal outcomes after metformin introduction

|  | n | Adjusted coefficients (95%CI) | p |
| --- | --- | --- | --- |
| **Birth weight (g)**  No GDM  GDM - Lifestyle changes  GDM – Insulin  GDM – Metformin | 8,047  117  186  69 | Reference  -132.5 (-209.5, -55.5)  -84.8 (-151.5, -17.9)  -131.4 (-230.0, -32.7) | 0.001  0.013  0.009 |
| **Head circumference (cm)**  No GDM  GDM – Lifestyle changes  GDM – Insulin  GDM – Metformin | 7,394  106  167  63 | Reference  0.2 (-0.06, 0.4)  0.2 (-0.02, 0.4)  0.1 (-0.2, 0.5) | 0.133  0.073  0.346 |
| **Mid-arm circumference (cm)**  No GDM  GDM – Lifestyle changes  GDM – Insulin  GDM – Metformin | 6,979  105  164  59 | Reference  -0.4 (-0.7, -0.2)  -0.2 (-0.4, -0.06)  -0.5 (-0.8, -0.2) | <0.001  0.009  0.001 |
| **Abdominal circumference (cm)**  No GDM  GDM – Lifestyle changes  GDM – Insulin  GDM – Metformin | 6,991  105  163  59 | Reference  -0.5 (-1.0, -0.06)  -0.6 (-1.0, -0.2)  -0.6 (-1.3, 0.009) | 0.029  0.005  0.053 |
| **Subscapular skinfold thickness (cm)**  No GDM  GDM – Lifestyle changes  GDM – Insulin  GDM – Metformin | 5, 560  85  135  51 | Reference  -0.3 (-0.5, -0.03)  -0.4 (-0.6, -0.1)  -0.3 (-0.6, 0.04) | 0.027  0.001  0.085 |
| **Triceps skinfold thickness (cm)**  No GDM  GDM – Lifestyle changes  GDM – Insulin  GDM – Metformin | 5,574  85  135  51 | Reference  -0.3 (-0.6, -0.1)  -0.2 (-0.5, -0.05)  -0.3 (-0.6, -0.05) | 0.004  0.014  0.020 |

*All models are adjusted for maternal BMI, height, age, ethnicity, parity, fasting and 2h glucose concentrations at OGTT, smoking, child sex, gestational age at birth and route of birth
